# Evaluation of the effectiveness of artificial intelligence models in radiopaque and radiolucent lesions of the maxillofacial region on panoramic radiographs

**DOI:** 10.1007/s11282-025-00838-x

**Published:** 2025-07-01

**Authors:** Zeynep Turanli Tosun, Nida Kumbasar, Muhammet Akif Sumbullu, Ozkan Miloglu

**Affiliations:** 1 Department of Oral, Dental and Maxillofacial Radiology, Faculty of Dentistry, Ataturk University, Yakutiye, 25240 Erzurum, Turkey; 2 TÜBİTAK, Informatics and Information Security Research Center (BİLGEM), 41470 İzmit, Kocaeli Turkey; 3 Department of Electrical Electronic Engineering, Faculty of Engineering, Ataturk University, 25240 Erzurum, Turkey

**Keywords:** Maxillofacial region, Panoramic radiography, Radiolucent lesion, Radiopaque lesion, Artificial intelligence, Deep learning

## Abstract

**Objectives:**

The aim of this study is to evaluate the success of algorithms used in deep learning (DL), a technique of artificial intelligence (AI), in the classification, detection, and segmentation of radiopaque, and radiolucent lesions in the maxillofacial region on panoramic radiographs (PR).

**Methods:**

This study included PRs of individuals aged 12 to 80 years who presented with radiopaque or radiolucent findings in the maxillofacial region based on radiological examination. Lesions were classified on the dataset obtained from the PRs using AlexNet, VGG16, and GoogleNet architectures. The location detection and segmentation of lesions were performed using the YOLOv8 architecture. The classification, object detection, and segmentation performances of the DL architectures were evaluated.

**Results:**

In the classification tasks using full PR, GoogleNet achieved the highest accuracy of 95.6%, with 97.1% precision and 95.5% F1 score in two-class lesion classification (lesion vs. no lesion). In distinguishing radiopaque and radiolucent lesions, VGG16 performed best, with 68.4% accuracy and 81.0% F1 score. For three-class and four-class classifications, GoogleNet again outperformed others with 61.6 and 75.7% accuracy, respectively. In cropped lesion-based classification, both GoogleNet and AlexNet achieved 96.5% accuracy. The YOLOv8m model demonstrated the best performance in object detection and segmentation, with 71.5% and 72.1% mean Average Precision (mAP), respectively.

**Conclusion:**

These findings suggest that DL architectures, particularly GoogleNet for classification and YOLOv8m for object detection and segmentation, demonstrate strong potential in the automated analysis of maxillofacial lesions on panoramic radiographs. Their high performance in distinguishing lesion types and accurately localizing pathological areas indicates that such models could assist clinicians in early diagnosis and treatment planning, potentially reducing reliance on more complex imaging methods.

## Introduction

Dental infections, cysts, neoplasms, dysplastic changes in the jaws, or metabolic diseases often cause radiopaque, and radiolucent lesions in the maxillofacial region. These lesions can lead to dental problems, phonation and functional deficiencies, and esthetic losses, while in some cases, they may persist without causing any symptoms in the patient. Bone pathologies may be incidentally detected in radiographs taken after clinical examination or identified through radiological methods guided by the patient’s complaints [1].

Radiological examination performed for diagnosis and treatment planning prior to dental treatments is a commonly used and essential step. The clinical and radiographic examination findings guide the clinician toward diagnosis. Frequently utilized techniques include intraoral methods such as periapical, occlusal, and bitewing radiographs, as well as extraoral methods like panoramic radiography (PR), which provides two-dimensional imaging. PRs, which are generally taken as initial radiographs, provide a broad imaging capability as they encompass both the upper and lower jaws simultaneously. Their greatest advantage is that it allows the detection of not only dental conditions but also many other pathological formations such as jaw lesions, temporomandibular joint degenerations, maxillary sinus diseases, and salivary gland stones [2]. It is practical as it provides a view of numerous anatomical structures of the maxillofacial region together in a single image. Additional advantages include ease of application, ergonomics in terms of patient cooperation, and a short imaging time. PR provides two-dimensional imaging and depth cannot be assessed during evaluation. The most significant disadvantages of this method include the superimposition of anatomical structures, distortion and magnification in the images, and lower resolution and detail compared to intraoral techniques. While it provides limited information due to its two-dimensional imaging capability, it is less expensive than advanced imaging techniques and is available in almost every clinic. Additionally, the amount of radiation exposure for patients is lower and the application time is shorter compared to advanced imaging techniques [3, 4].

Artificial intelligence (AI) refers to systems capable of generating thought through machines by mimicking human intelligence [5]. There are numerous studies on AI in the field of radiology [6–10]. Among the aims of the widespread adoption of this technology are reducing diagnostic time, the need for specialists, and human errors, while enabling AI-assisted software to detect certain findings that may go unnoticed by the human eye. For this purpose, AI can be used in radiological examinations to diagnose diseases even before clinical symptoms appear. AI developed for radiological evaluations can assist dentists and dentomaxillofacial radiologists in assessment and interpretation [11].

Previous studies have demonstrated the potential of deep learning (DL) models in detecting specific pathologies such as odontogenic cysts, tumors, or maxillary sinus lesions using panoramic radiographs. However, many of these investigations have focused on isolated lesion types, limited anatomical regions (e.g., only the mandible), or small, histologically confirmed datasets. Few have explored the simultaneous classification and localization of both radiopaque and radiolucent lesions across the full maxillofacial region in a diverse, real-world PR dataset. Moreover, te comparative analyses of multiple DL architectures across different classification setups (e.g., full-image vs. cropped lesion areas) remain limited in the literature [12–16].

Our study aims to bridge this gap by evaluating the diagnostic performance of three CNN-based classification models (AlexNet, VGG16, GoogleNet) and five YOLOv8 object detection models on PR. By comparing the classification on full PRs and cropped lesion regions, and by addressing both radiolucent and radiopaque lesion types, this study provides a more comprehensive analysis of AI capabilities in routine dental imaging.

Detecting and characterizing radiopaque and radiolucent lesions in the maxillofacial region can be particularly challenging due to the overlap of anatomical structures and variations in lesion presentation. Early and accurate diagnosis is critical to prevent potential complications, such as lesion progression, unnecessary surgical interventions, or missed malignant transformations. In routine dental practice, however, access to advanced imaging techniques is often limited, and diagnostic interpretation can vary depending on clinician experience. Given these limitations, the integration of AI-based systems in panoramic radiograph analysis may offer a reliable and accessible solution to assist clinicians in early detection and classification of lesions.

Therefore, this study hypothesizes that DL algorithms can accurately detect, classify, and segment radiopaque and radiolucent lesions on PRs. The goal is to evaluate their diagnostic performance and highlight their potential for integration into routine clinical workflows, where they may contribute to faster, more objective, and cost-effective patient care.

## Methods

This study’s compliance with scientific ethical principles was approved by the Ethics Committee of Ataturk University, Faculty of Dentistry, Faculty of Dentistry, with the decision dated 02/12/2022, numbered 11/2022 and 89, and the study was conducted in accordance with the principles of the Declaration of Helsinki. 

In our study, the detection, classification, and segmentation of radiopaque and radiolucent lesions in the maxillofacial region on PRs were addressed using convolutional neural network (CNN)-based DL models by utilizing archive records. During this process, a supervised learning approach, which builds models with labeled data, was adopted. Thus, the models were enabled to predict outputs for previously unseen new input data.

The study included PRs of individuals aged 12 to 80 years who visited the Department of Oral, Dental, and Maxillofacial Radiology at Ataturk University, Faculty of Dentistry, either for the detection of existing lesions in the maxillofacial region or who were incidentally found to have lesions during routine radiographic examinations. PR (Planmeca Promax 2D, Panoramic System, Helsinki, Finland) images of these patients were downloaded in Windows Bitmap (.bmp) format. Patients under 12 years of age, patients with congenital syndromes, craniofacial syndromes, cleft lip and palate, craniofacial bone diseases, patients with pathologies in the craniofacial region, or those who had undergone orthognathic surgery, as well as PR images with patient or radiological imaging artifacts (metal artifacts, artifacts resulting from the patient’s position during acquisition, movement artifacts, etc.), were excluded from the review. A total of 650 lesions, including 415 radiolucent and 235 radiopaque, were evaluated by an observer undergoing specialty training on 500 PRs. Additionally, 500 healthy PRs without lesions were included in the study to enable lesion presence-absence classification. Ultimately, the experimental studies were conducted with 1000 PRs. The success of AI algorithms in classifying lesions, detecting their locations within bounding boxes, and pixel-based segmentation was evaluated. 

For lesion segmentation on PRs, we used Make Sense AI (v1.11.0-alpha, GPL-3.0 License), a freely available, browser-based image annotation tool widely adopted in computer vision research [17]. This platform was selected due to its ease of use, support for polygon-based annotations, and ability to export labels in multiple formats compatible with common DL frameworks (e.g., YOLO, COCO, JSON). Its visual interface allows for precise, manual delineation of irregular shapes, which is especially important for outlining complex lesion contours.

**Fig. 1:**
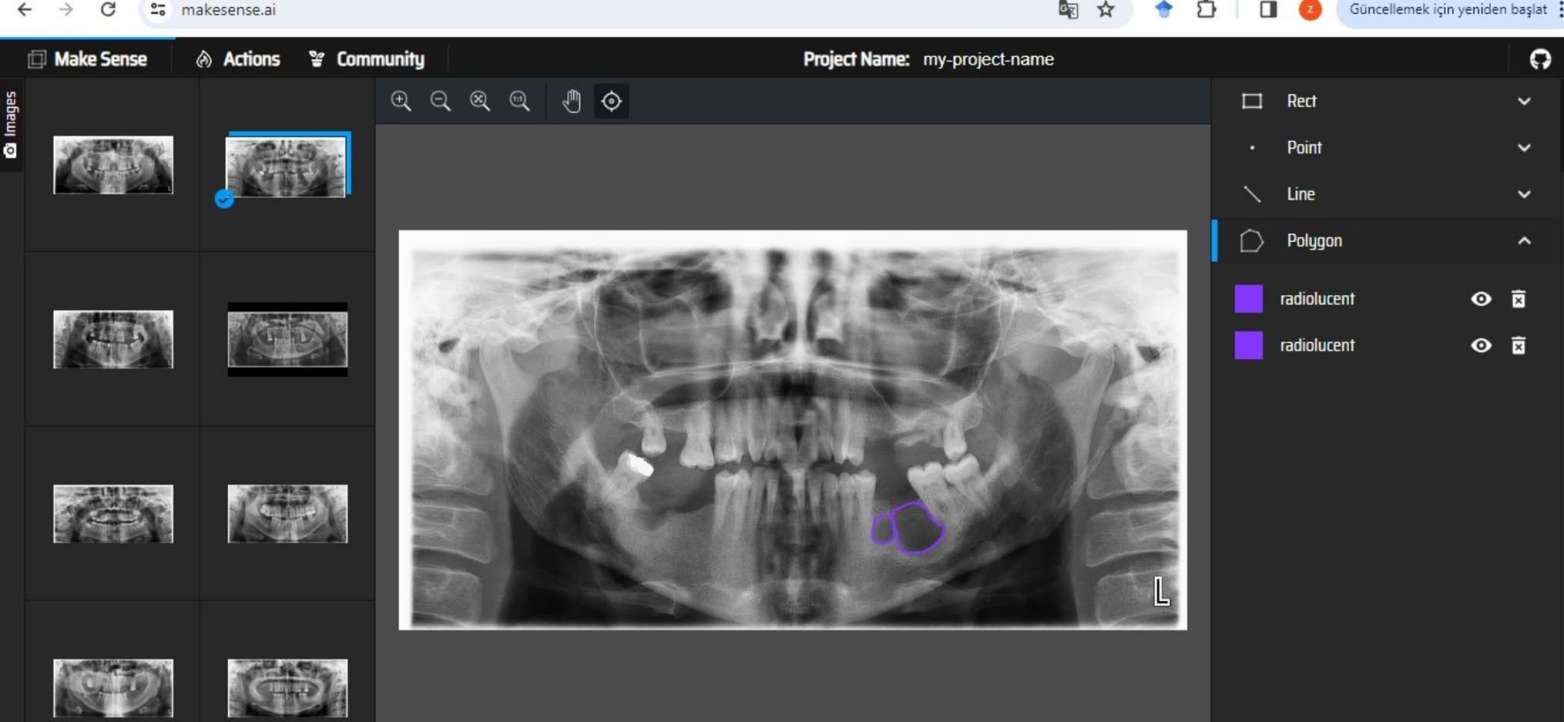
Labeling process of a radiolucent lesion on PR using the Makesense AI program

**Fig. 2:**
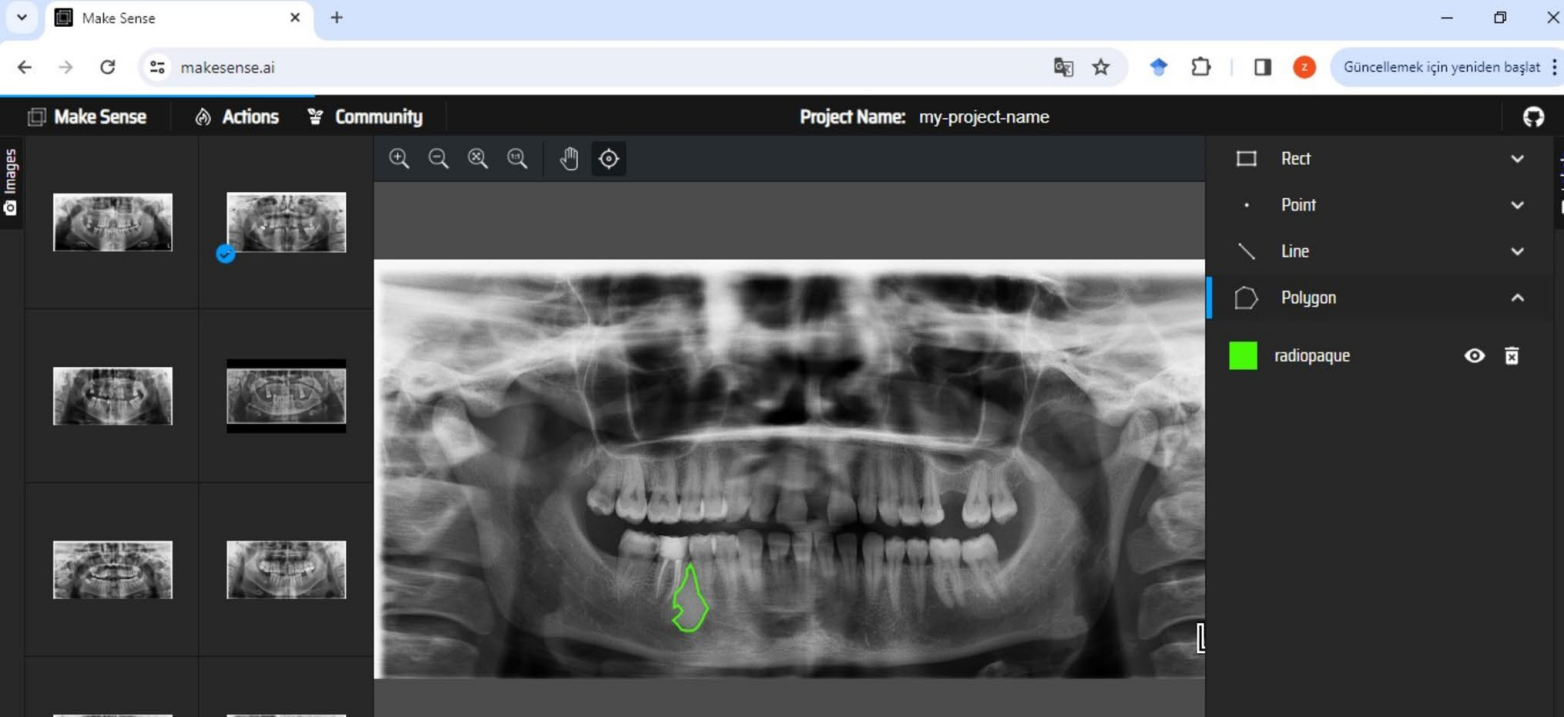
Labeling process of a radiopaque lesion on PR using the Makesense AI program

All annotations were conducted manually by an oral and maxillofacial radiology specialist. Each lesion was outlined using a polygon tool, with different colors assigned to radiopaque and radiolucent lesion types to maintain class consistency and aid visual review (Figs. 1 and 2). The resulting label files, saved in YOLO format, contained coordinate data that were used both for training YOLOv8 segmentation models and for deriving bounding boxes in object detection tasks. The manual labeling process ensured accurate ground truth masks, which are essential for evaluating pixel-level model performance and for training instance segmentation architectures effectively.

All experimental studies were conducted using Python and MATLAB programming languages on a 64-bit Ubuntu 18.04 system with 128 GB of random access memory and two graphics processing units (GeForce RTX 2080 TITAN; NVIDIA).

### Creation of datasets

In this study, three separate datasets were created to determine radiopaque and radiolucent lesions in the maxillofacial region using DL models, with the aim of classifying the presence or absence of lesions on PRs, defining their localization using bounding boxes, and performing pixel-based image segmentation, thereby employing three different approaches to the problem.

The main objective of the study is to determine the location and pixel-wise boundaries of the lesioned area on the whole PR. However, in a preliminary stage, it is planned to analyze the characteristic discrimination of the lesions. For this, lesion-containing regions were cropped from the PR, eliminating off-target areas of the PR. Subsequently, the effect of the lesioned region on the character of the PR was studied.

#### Classification

Classification was performed in two ways:

1. The presence of radiopaque or radiolucent lesions was assessed across the entirety of the PRs. This classification included four categories.

Classification by giving the entire PR

**Fig. 3:**
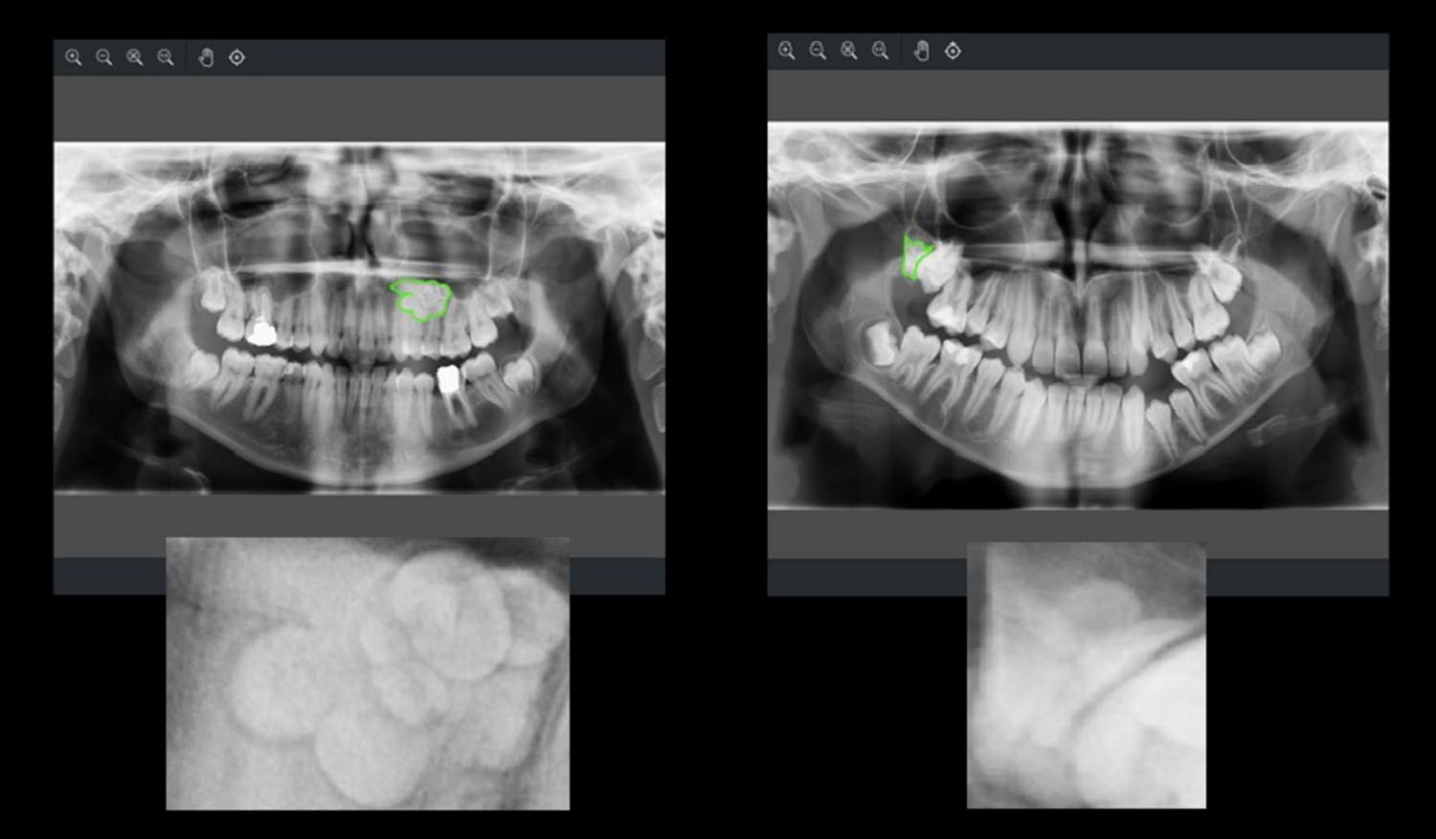
Data samples of cropped radiopaque lesions

**Fig. 4:**
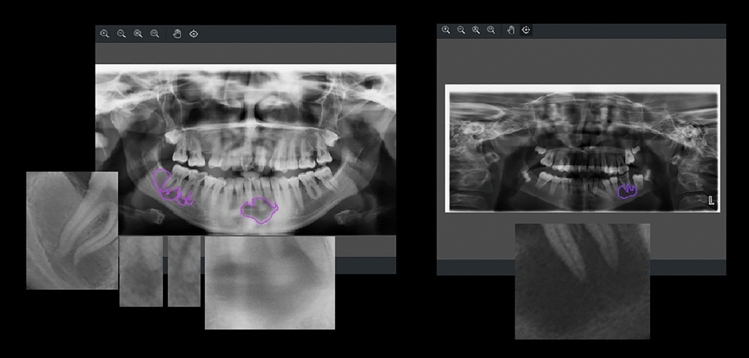
Data samples of cropped radiolucent lesions

- There is a lesion - No lesion (2 classes)
- Radiopaque-Radiolucent (2 classes)
- Radiopaque-Radiolucent-Radiopaque/Radiolucent (3 classes)
- Radiopaque-Radiolucent-Radiopaque/Radiolucent- No lesion (4 classes)

2. The distinctiveness of radiopaque and radiolucent lesions was examined by trimming the lesion area in the entire PR (Figs. 3 and 4). During the data pre-processing stage, the bounding box coordinates were automatically determined based on segmentation labels in the PRs. Subsequently, the regions within the bounding boxes were cropped from the PRs and saved as new images for this problem.

AlexNet [18], VGG16 [19], and GoogleNet [20] DL models were employed for all classification tasks due to their established effectiveness in medical image analysis and their architectural diversity, which enables comparative evaluation. AlexNet, with its eight-layer structure, was among the first CNNs to demonstrate strong performance on large-scale image classification tasks. VGG16 is a deeper network consisting of 16 weight layers and is known for its uniform architecture using small 3 × 3 filters, which improves feature extraction. GoogleNet (Inception v1) introduces inception modules that allow multiple convolution operations at different scales to be performed simultaneously, making it computationally efficient and effective for capturing both global and local patterns.

All three models were implemented using a transfer learning approach with pre-trained ImageNet weights and fine-tuned on the study dataset. Many experiments were carried out during the fine tuning process. The most successful performance parameters were mentioned. During training, the learning rate was set to 0.001, the batch size was 32, and training was performed for 50 epochs using the Adam optimizer. Data augmentation techniques such as horizontal flipping, rotation (±15°), and brightness variation were applied to increase generalizability and reduce overfitting.

A 10-fold cross-validation method was used to assess model performance. The dataset was divided into 10 equal parts, with 9 parts used for training and the remaining part for testing, rotated sequentially. The average results across folds were calculated to provide a robust estimate of model accuracy and to minimize variance due to sample distribution.

#### Object detection and instance segmentation

**Fig. 5:**
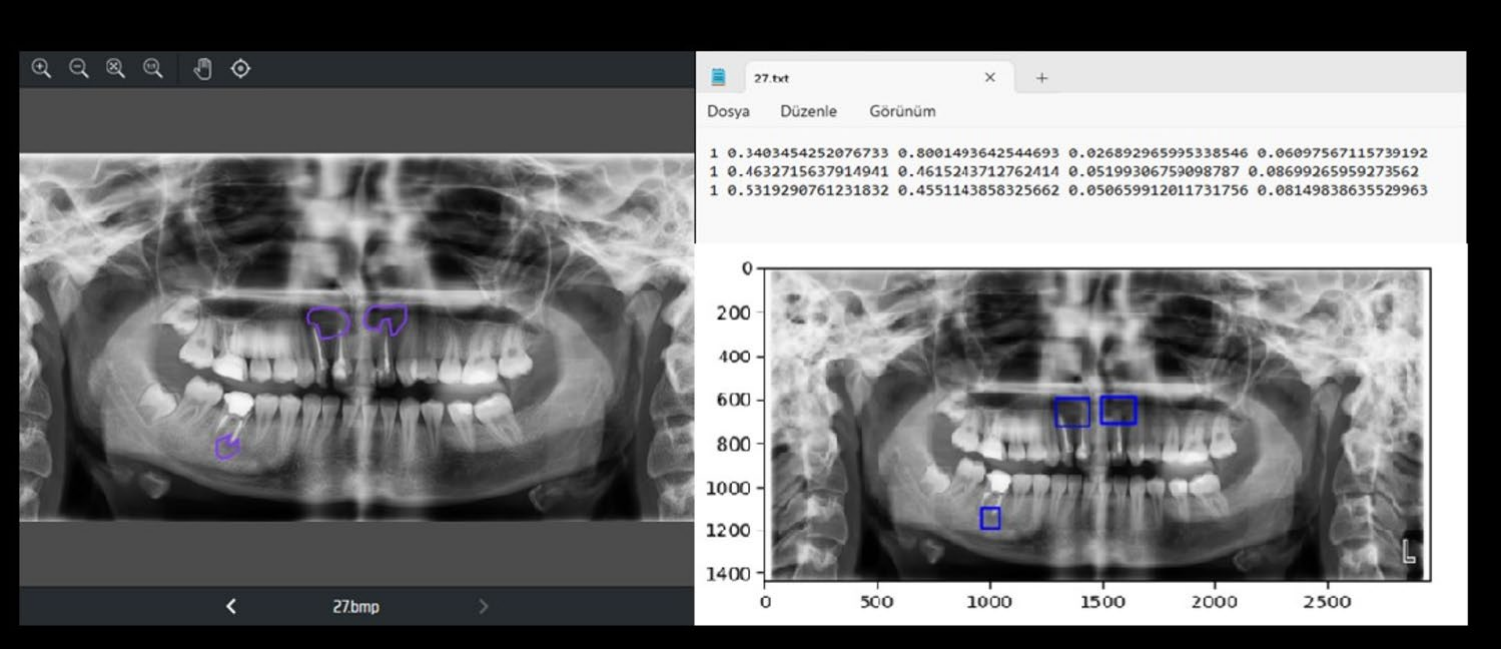
Example of a label file prepared for object detection along with PR

This problem focused on identifying the locations of regions on the entire PR with radiopaque or radiolucent lesions and determining which pixels constituted the lesion area. Label files containing lesion areas labeled with the Makesense AI program [17] for object detection and segmentation were prepared (Fig. 5). In the experimental studies, five different architectures of the CNN-based DL model YOLOv8 [21, 22], differing in depth and named nano (n), small (s), medium (m), large (l), and extra-large (xl), were used. For pixel-based segmentation of the lesion area, a total of 500 PRs comprising the dataset were randomly divided as follows: 350 (70%) as training data, 75 (15%) as validation data, and 75 (15%) as test data. Table 1 shows the distribution of radiopaque and radiolucent lesions in the training, validation, and test datasets.

**Table 1:** Data distribution in object recognition problem

Lesion area	Train	Validation	Test	Totally
Number of PR	350	75	75	500
Radiopaque	170	26	39	235
Radiolucent	295	62	58	415
Totally	465	88	97	650

### Statistical analysis

PRs were examined by a researcher (Z.T.T) with 5 years of experience in oral and maxillofacial radiology. In order to assess the intra-observer reliability of the study, 100 randomly selected PRs were re-analyzed by the same observer one month later. There was excellent agreement between the first and second measurements, with an intraclass correlation coefficient (ICC) value of 0.99. In addition, to increase the reliability of the study, these 100 randomly selected PRs were evaluated by a second observer (M.A.S.) with 21 years of experience in oral and maxillofacial radiology). In the evaluation, a complete agreement was achieved in the inter-observer agreement. In the Cohen’s Kappa test, this ratio was found to be approximately 1.0. This result shows high agreement between the two observers.

## Results

In this study, the AI success of classification problems was evaluated through Accuracy, Precision, Recall and F1 Score (Eqs. 1–4) metrics.

1 $$Accuracy=\frac{TP+TN}{TP+TN+FP+FN}$$

2 $$Precision=\frac{TP}{TP+FP}$$

3 $$Recall=\frac{TP}{TP+FN}$$

4 $$F1 score=2\frac{Precision.Recall}{Precision+Recall}$$

The abbreviations in the mentioned equations correspond to true positive (TP), true negative (TN), false positive (FP), and false negative (FN) values in the confusion matrix.

Mean Average Precision (mAP), visualized by a precision-recall plot, was used to evaluate the performance of the models in object detection and segmentation problems. mAP is used to evaluate the precision of the model’s predictions across different classes. It is computed by calculating the average precision (AP) (Eq. 5) for each class and then averaging these values. The AP for each class is determined by plotting precision-recall curves and calculating the area under the curve. We present the mAP (Eq. 6) values for each model in our results. In this study, the threshold intersection over union between the prediction and target frames was set to a value 0.5.

5 $$\text{AP}@\text{threshold}=\int p(r)dr$$

6 $$\text{mAP}@\text{threshold}=\frac{1}{n}\sum_{i=1}^{n}{AP}_{i}\text{ for n classes}$$

### Classification results

In the first approach to the classification problem in our study, the success of three separate DL architectures of AlexNet, VGG16, and GoogleNet was investigated by fine-tuning them with our dataset to determine the presence of radiopaque and radiolucent lesions on the entirety of the PRs. The results showed that the proportional performance of the AlexNet model in lesion presence-absence classification was 93.4% accuracy, 92.7% precision, 94.2% sensitivity, and 93.4% F1 score; the VGG16 model demonstrated a proportional performance of 64.7% accuracy, 63.3% precision, 69.6% sensitivity, and 66.2% F1 score; and the GoogleNet model achieved 95.6% accuracy, 97.1% precision, 94% sensitivity, and 95.5% F1 score. According to this, in the binary classification of lesion presence or absence across the entirety of PRs, the most successful model was GoogleNet, while the lowest success rates were obtained with VGG16 (Table 2).

**Table 2:** Comparison of methods used in classifying lesions as present or absent

	Accuracy (%)	Precision (%)	Sensitivity (%)	F1 score (%)
AlexNet	93.4	92.7	94.2	93.4
VGG16	64.7	63.3	69.6	66.2
GoogleNet	95.6	97.1	94.0	95.5

The second approach to classification across the entirety of PRs involves distinguishing between radiopaque and radiolucent lesions. According to the obtained data, the proportional performance of the AlexNet model in radiopaque-radiolucent lesion classification was 63.7% accuracy, 70.6% precision, 80.8% sensitivity, and 75.3% F1 score; the proportional performance of the VGG16 model was 68.4% accuracy, 68.8% precision, 98.8% sensitivity, and 81.0% F1 score; and the GoogleNet model demonstrated a proportional performance of 64.1% accuracy, 70.5% precision, 82.0% sensitivity, and 75.8% F1 score. According to this, the highest success rates in radiopaque-radiolucent lesion classification across the entirety of the PRs were achieved with the VGG16 model (Table 3).

**Table 3:** Comparison of methods used in the classification of radiopaque and radiolucent lesions

	Accuracy (%)	Precision (%)	Sensitivity (%)	F1 score (%)
AlexNet	63.7	70.6	80.8	75.3
VGG16	68.4	68.8	98.8	81.0
GoogleNet	64.1	70.5	82.0	75.8

The third approach to classification across the entirety of the PRs involved a three-class problem in which the presence of radiopaque, radiolucent, and radiopaque-radiolucent lesions was determined. According to the obtained data, the proportional performance of the AlexNet model in the three-class problem was 55.6% accuracy, 66.2% precision, 71.9% sensitivity, and 68.9% F1 score, while the VGG16 model demonstrated 58% accuracy, 65% precision, 80.2% sensitivity, and 71.8% F1 score. The proportional performance of the GoogleNet model in this three-class problem was found to be 61.6% accuracy, 65.3% precision, 88.3% sensitivity, and 75 F1 score, with GoogleNet achieving the highest success in this problem (Table 4).

**Table 4:** Comparison of methods used in the classification of radiopaque, radiolucent and radiopaque-radiolucent lesions

	Accuracy (%)	Precision (%)	Sensitivity (%)	F1 score (%)
AlexNet	55.6	66.2	71.9	68.9
VGG16	58.0	65.0	80.2	71.8
GoogleNet	61.6	65.3	88.3	75.0

The final approach to the problem of lesion classification across the entirety of the PRs involved a four-class problem. According to the obtained data, the proportional performance of the AlexNet model in the four-class problem was 68.3% accuracy, 92.4% precision, 84.6% sensitivity, and 88.3 F1 score, while the VGG16 model demonstrated 70.6% accuracy, 82% precision, 88.6% sensitivity, and 85.1 F1 score. The proportional performance of the GoogleNet model in this four-class problem was found to be 75.7% accuracy, 92.2% precision, 99.2% sensitivity, and 95.5% F1 score, with GoogleNet achieving the highest success in this problem (Table 5).

**Table 5:** Comparison of methods used in the classification of radiopaque, radiolucent, radiopaque-radiolucent and no lesion

	Accuracy (%)	Precision (%)	Sensitivity (%)	F1 score (%)
AlexNet	68.3	92.4	84.6	88.3
VGG16	70.6	82.0	88.6	85.1
GoogleNet	75.7	92.2	99.2	95.5

In our study, the second approach to the classification problem focused on the differentiation of radiopaque and radiolucent lesions by cropping only the lesion-containing regions of the PRs. The results showed that in the binary classification performed within the cropped lesion areas of the PRs based on lesion type, the proportional performance of AlexNet was 96.5% accuracy, 95.9% precision, 96.4% sensitivity, and 96.2% F1 score; VGG16 demonstrated 94% accuracy, 93.8% precision, 93% sensitivity, and 93.4% F1 score; and GoogleNet exhibited 96.5% accuracy, 96.4% precision, 95.8% sensitivity, and 96.1% F1 score (Table 6).

**Table 6:** Comparison of methods used in binary classification according to lesion type within cropped lesion areas in panoramic radiographs

	Accuracy (%)	Precision (%)	Sensitivity (%)	F1 score (%)
AlexNet	96.5	95.9	96.4	96.2
VGG16	94.0	93.8	93.0	93.4
GoogleNet	96.5	96.4	95.8	96.1

### Object detection: instance segmentation results

The comparison of performance success for YOLOv8 (n-s-m-l-xl) sub-models in localizing and segmenting radiopaque or radiolucent lesion regions on PR is presented in Table 7.

**Table 7:** Comparison of object detection - instance segmentation YOLOv8 models

	Radiopaque		Radiolucent		Overall performance	
	Box	Mask	Box	Mask	Box	Mask
YOLOv8n	47.1	50.6	58.3	58.3	52.7	54.5
YOLOv8s	57.2	57.0	65.2	60.9	61.2	59.0
YOLOv8m	67.6	65.8	75.4	78.4	71.5	72.1
YOLOv8l	57.0	55.4	77.8	74.1	64.4	64.8
YOLOv8xl	61.4	54.3	71.8	73.5	66.6	63.9

**Fig. 6:**
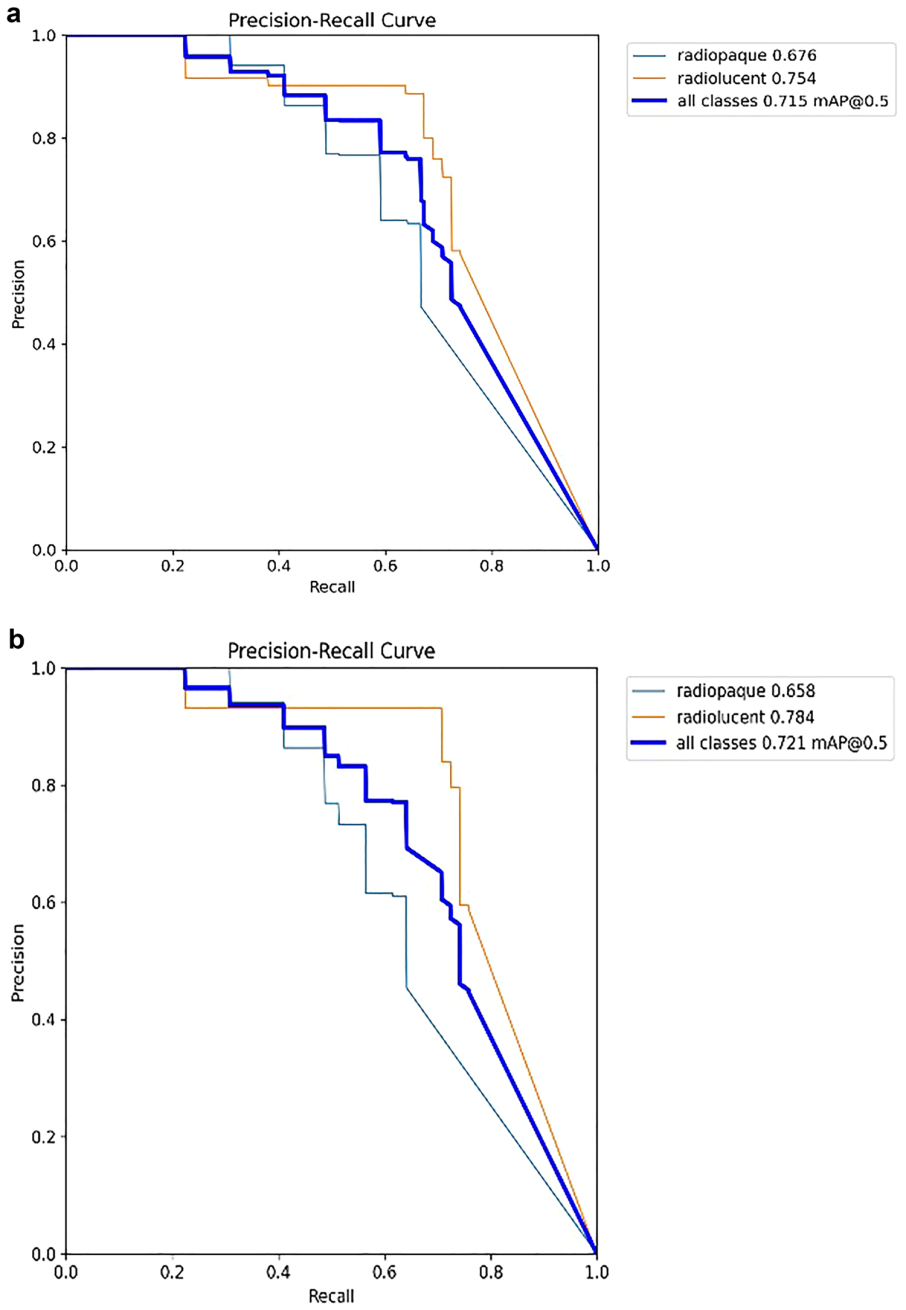
YOLOv8m (Top) bounding box (bbox) analysis results and (Bottom) mask analysis results

**Fig. 7:**
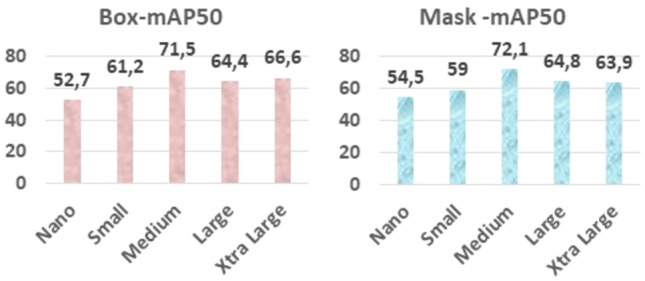
Graph comparing performance based on YOLOv8 architectures

**Fig. 8:**
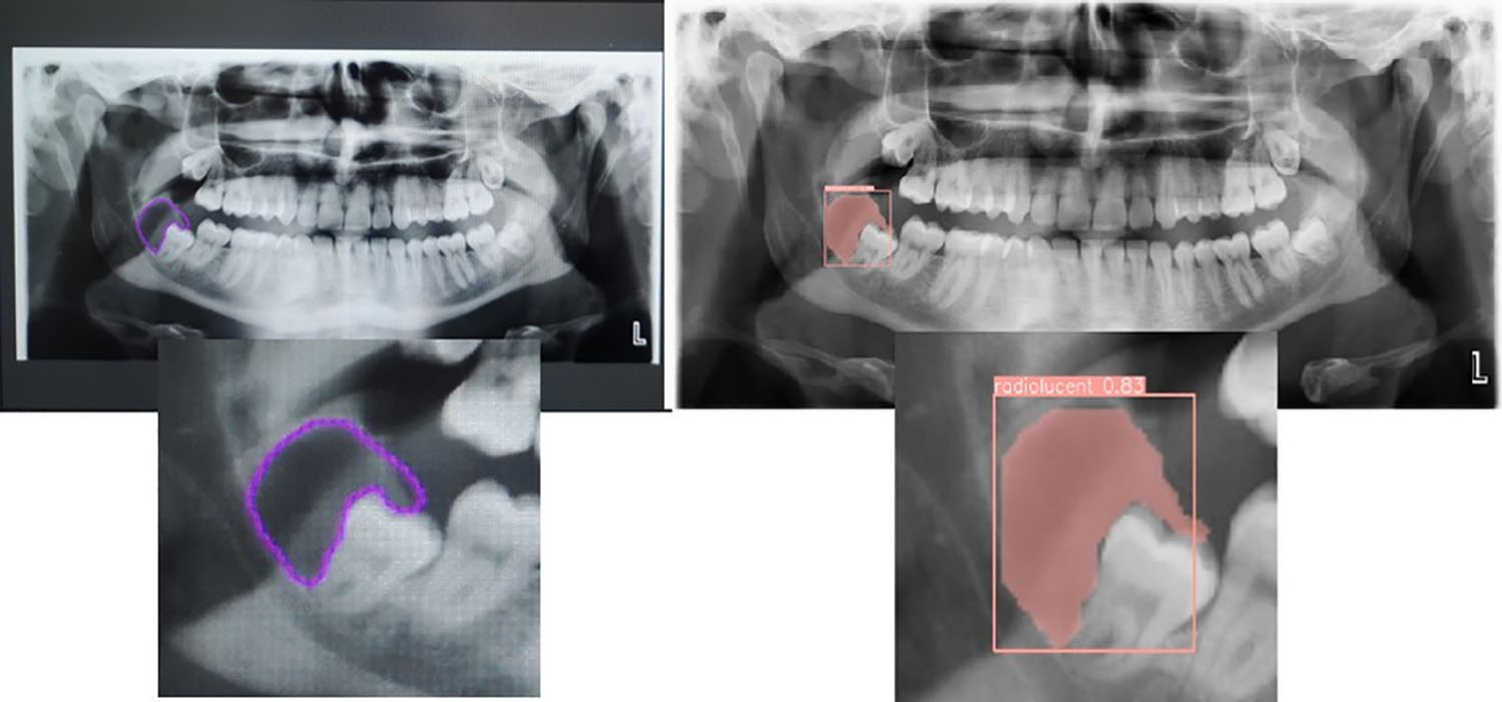
On the right, the region of radiolucent lesions annotated by the expert; on the left, the region of radiolucent lesions predicted by the YOLOv8m model

**Fig. 9:**
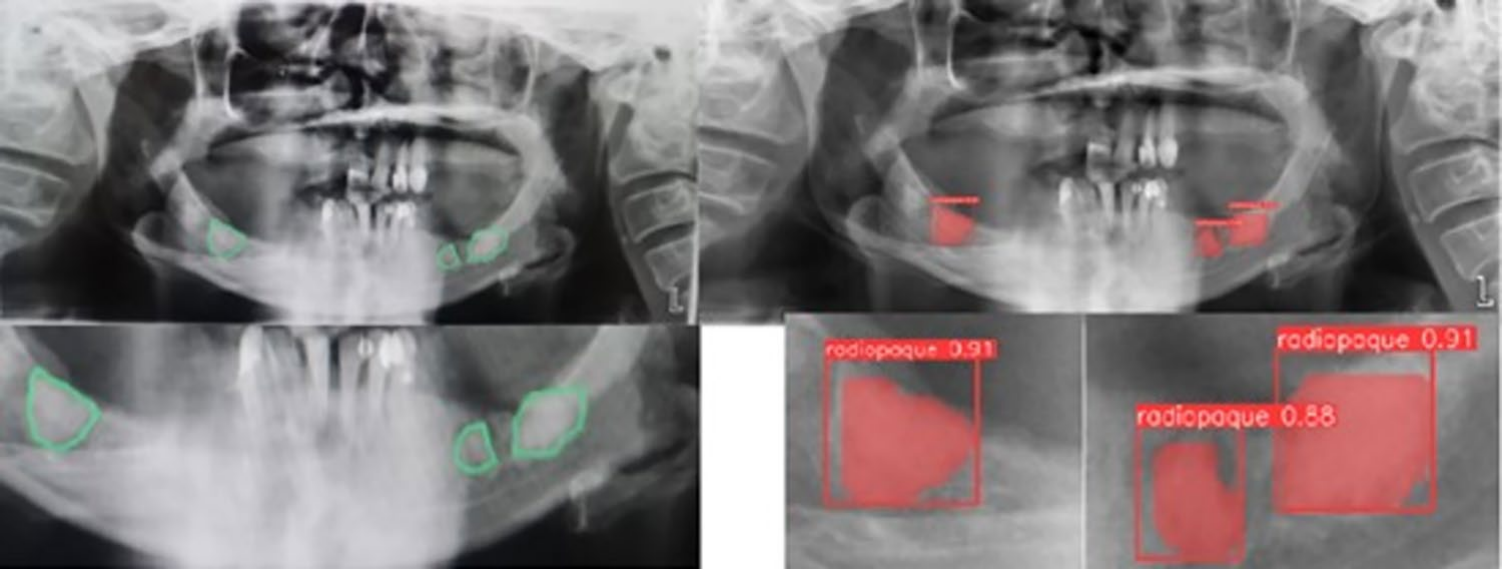
On the right, the region of radiopaque lesions annotated by the expert; on the left, the region of radiopaque lesions predicted by the YOLOv8m model

**Fig. 10:**
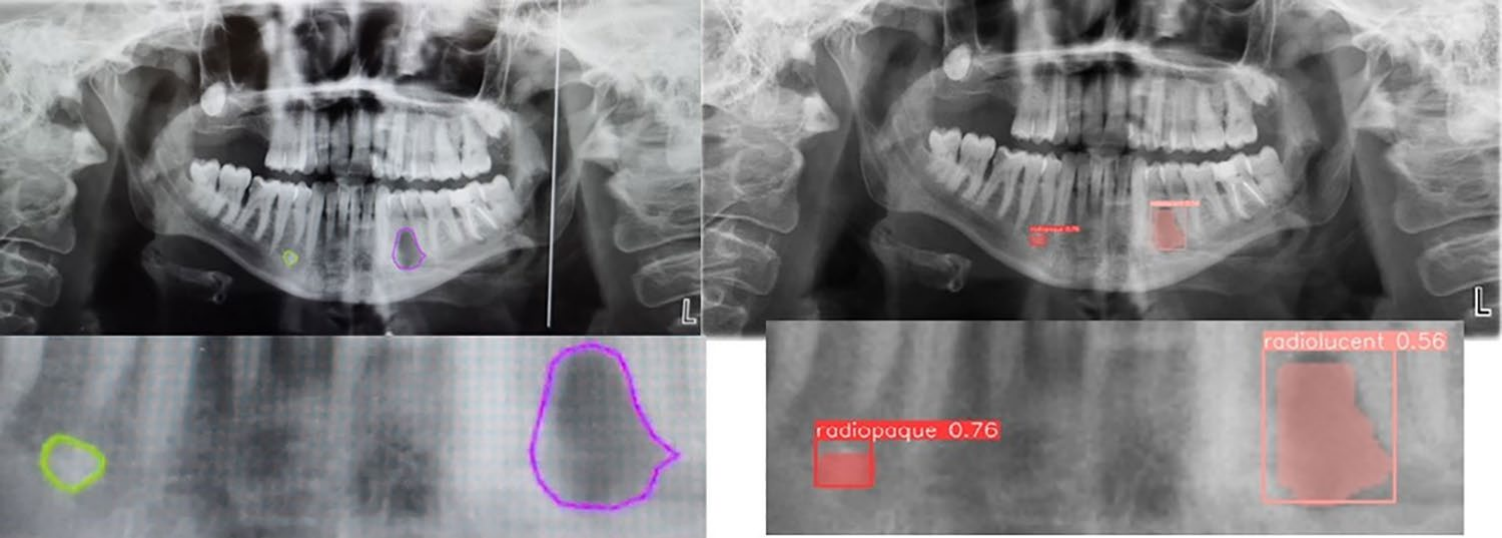
On the right, the region with radiopaque and radiolucent lesions labeled by the expert; on the left, the region with radiopaque and radiolucent lesions predicted by YOLOv8m

Based on the obtained data, in the segmentation study conducted to detect radiopaque and radiolucent lesions across the entirety of PRs and define them at the pixel level, the YOLOv8 architecture was fine-tuned and used and the YOLOv8m architecture demonstrated the best performance both in bounding box detection, which involves enclosing the identified object within a box, and in pixel-level classification, that is, mask values (Fig. 6a, b, and Fig. 7). In the same study, YOLOv8n exhibited the lowest success in both groups. Sample outputs from the YOLOv8m model are shown in detail in Figs. 8, 9, and 10. The mentioned figures feature enlarged views of the lesion region on the PR, placed beneath the images to allow for better analysis of the relevant region by the reader.

Especially at this stage to enhance the interpretability of CNN and to better understand the decision-making process, we employed the gradient-weighted class activation mapping (Grad-CAM) technique [23]. Grad-CAM generates visual explanations by highlighting the regions in the input image that are most influential in the model’s prediction. Specifically, it utilizes the gradients of the target class flowing into the final convolutional layer to produce a coarse localization map that highlights important areas for classification.

**Fig. 11:**
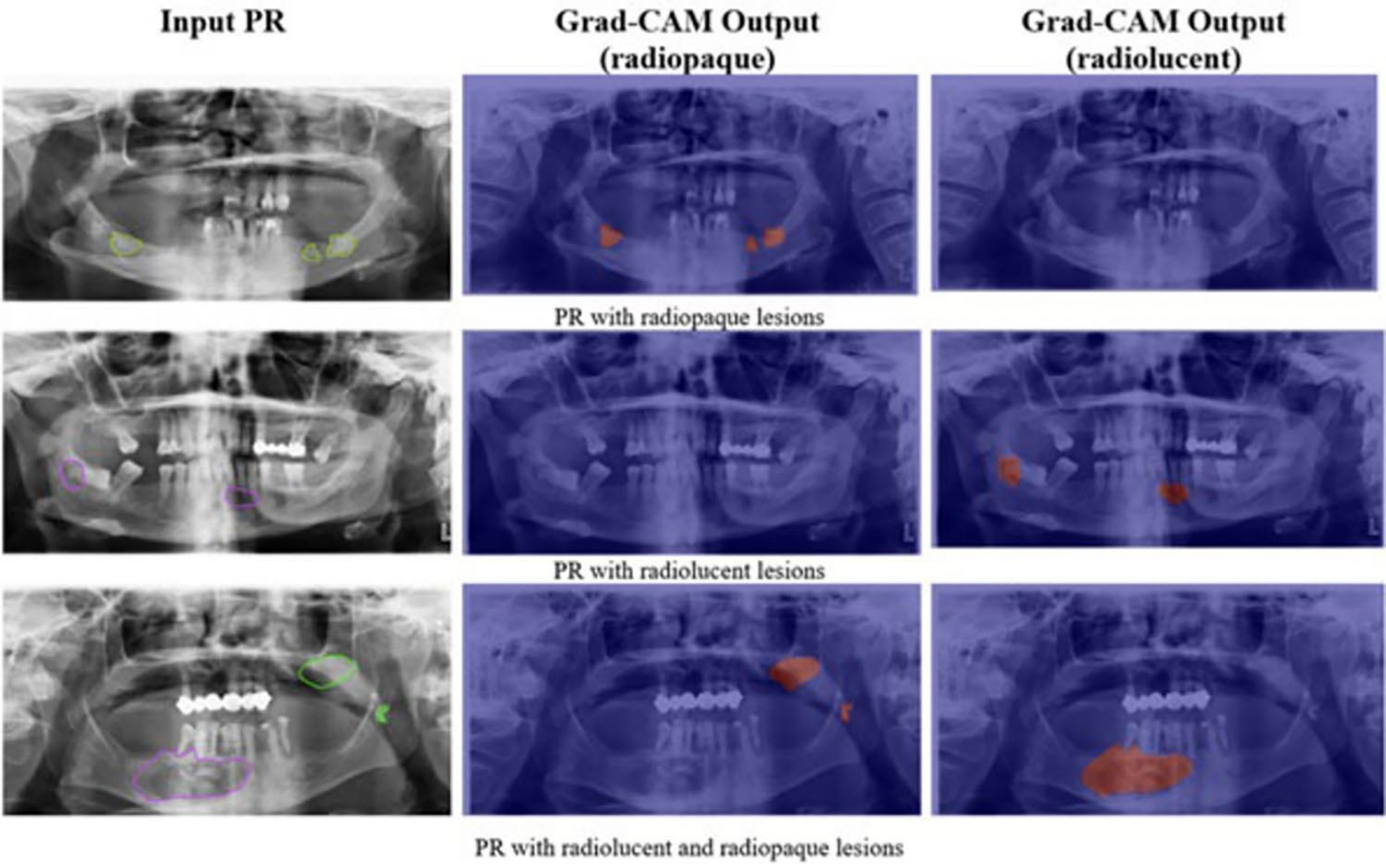
Successful segmentation examples using Grad-CAM outputs from YOLOv8m model

**Fig. 12:**
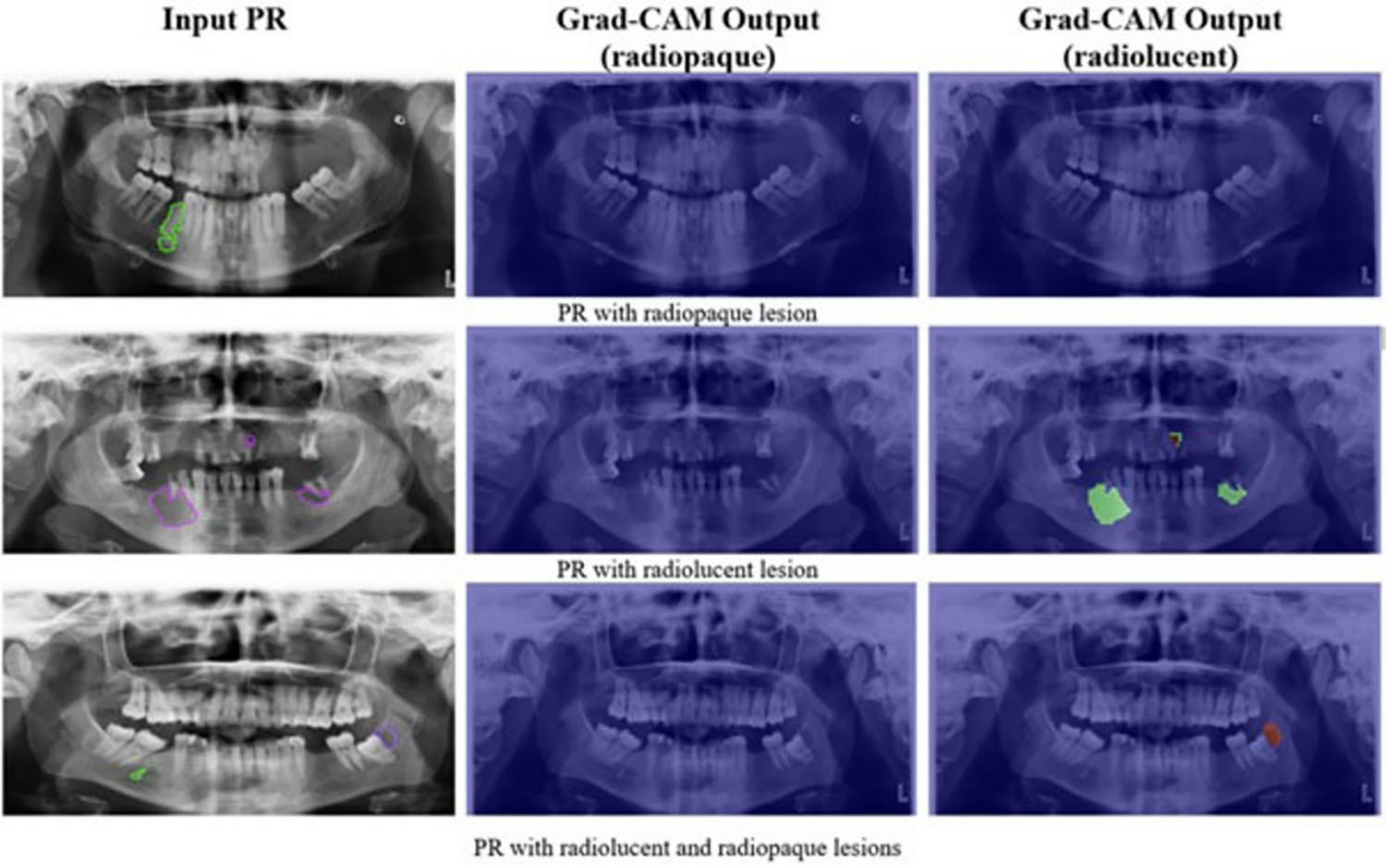
Failed segmentation examples using Grad-CAM outputs from YOLOv8m model

In our visualizations, we applied the widely-used jet-colormap, where warmer colors (e.g., red, yellow) indicate regions with higher importance, and cooler colors (e.g., blue, green) correspond to areas with less contribution to the decision. These heatmaps demonstrate that the model predominantly focuses on semantically meaningful features relevant to the target class. For instance, in correctly classified cases, the model consistently emphasizes anatomical or structural features aligned with expert interpretation. Furthermore, we analyzed several misclassified cases through Grad-CAM to explore whether the model’s focus was misplaced or if the ambiguity was due to overlapping class characteristics. For the segmentation problem, which is the main motivation, Grad-CAM outputs were obtained using the YOLOv8m trained model with the highest performance. Figure11 shows the results where the model succeeds, while Fig. 12 illustrates the images where the model fails.

## Discussion

The maxillofacial region houses various tissues, such as teeth, bone tissue, cartilage tissue, nerves, and vascular structures, making it an anatomical area where pathological formations originating from these tissues are frequently observed. Due to the diversity and origins of these pathological formations from different tissues, various classifications have been made based on their etiology, clinical appearance, histopathological features, and radiographic images. The diversity of cysts, tumors, and tumor-like lesions occurring in the maxillofacial region, along with their similar clinical and radiological characteristics, makes diagnosis, and classification particularly challenging for clinicians [24].

With the use of X-rays in dental radiology, the diagnosis and identification of lesions have become easier through radiological evaluations in addition to clinical examinations. The first method commonly used in radiological assessment is direct radiography, and PRs are the most frequently preferred, enabling the simultaneous observation of teeth, supporting tissues, and most structures in the facial region. However, due to disadvantages such as the inability to visualize lesions in different planes, magnifications, geometric distortions, superimpositions, and low resolution, advanced imaging methods are often required. The techniques such as computed tomography, magnetic resonance imaging, and ultrasonography, however, are costly and may not be available in every facility. Additionally, interpreting the obtained images requires specialist physicians and experience. Therefore, the application of AI in clinical and radiological practice for the automatic diagnosis of lesions holds significant value [25]. In this study, the performance of AI models, which can learn from larger datasets, in detecting lesions in the maxillofacial region was investigated using PRs, which are frequently utilized in clinical practice.

In the literature, studies on the use of AI in dental radiology show that the image materials forming the dataset typically consist of PRs and, less frequently, cone-beam computed tomography (CBCT) images [11]. The use of PRs, which are common in radiological diagnosis, provides an advantage for AI techniques to better learn lesion characteristics. This can enable the early detection of lesions and reduce the need for advanced imaging.

Although radiological images include numerous anatomical structures that need to be scanned and evaluated, AI applications can improve the accuracy of diagnosing complex pathological formations [26]. In a study by Yang et al. [12], a DL method was developed that can detect and classify lesions in four different categories observed in the maxilla and mandible using PRs. This study is the first known to include datasets from both the maxilla and mandible. In this study, the YOLOv8 architecture and a dataset comprising specific histopathologically confirmed lesions were used as the method. In our study, in addition to the entirety of the maxilla and mandible, a broader area of the facial region on PRs was also included in the dataset. The lesions targeted for detection were evaluated based on the densities they produced in radiological images rather than their histopathological characteristics. Despite including a smaller dataset, the accuracy rate of object detection in general classification using the YOLOv8 architecture in our study was found to be higher compared to the study by Yang et al [12]. We believe this is due to the examination of a broader anatomical area and the increased diversity achieved by labeling across more regions.

In another study with a dataset consisting of 226 PRs [13], the performance of the YOLOv8 DL architecture in detecting and segmenting radiolucent lesions in the mandible was investigated. Using the YOLOv8l sub-model, results without data augmentation showed mAP50 values of 75.8% for object detection and 75.1% for segmentation. In the same study, experiments conducted with data augmentation achieved success rates of 97.5% and 96.6% for object detection and segmentation, respectively. When compared to the proposed study, in our study, using the YOLOv8m architecture, which yielded the highest performance in the experiments, results obtained without increasing the data size showed values of 75.4% for object detection and 78.4% for segmentation mask prediction exclusively for radiolucent lesions. We believe that the relatively higher success rates obtained from our study compared to this study are due to the inclusion of datasets from both jaws and a larger number of data samples.

In a study conducted by Ariji et al. [14], five different radiolucent lesions located exclusively in the mandible, which are relatively more common, were detected and classified on PRs using DL methods. For this purpose, a dataset consisting of 210 PRs was utilized. The highest success rate in object detection was achieved for dentigerous cysts at 88%, while the success rate for classification was 82%. In the aforementioned study, radiolucent and radiopaque lesions were analyzed using AI models solely based on their radiological density, without differentiation between jaws or histological classification. Ultimately, a sensitivity of 98.8% was observed in binary classification. In our study, a more fundamental approach was taken to the problem by evaluating the presence or absence of lesions across the entirety of PRs, and the method we used distinguished 470 out of 500 lesions, achieving an accuracy rate of 95.6%. We believe that the higher success rates in object detection and classification in our study compared to those of Ariji et al. [14] are due to the inclusion of both jaws and a larger dataset in the study.

The failure in detecting and classifying lesions is often due to lesions that are small in size, have poorly defined boundaries, and produce weak radiological images. Particularly, lesions that are challenging to diagnose even for experienced clinicians, due to unclear early-stage pathologies and the superimposition of surrounding anatomical structures, can pose significant challenges for the AI algorithms used. For this reason, in this study, lesion classification was conducted both across the entirety of PRs and by cropping the lesion areas, revealing notable differences between the two approaches. The highest success rate for the classification of radiopaque and radiolucent lesions across the entirety of PRs was achieved with the VGG16 model at 68.4%, whereas for cropped images, this rate significantly increased, reaching up to 97.7%. To achieve higher success rates and ensure the models are less affected by pattern differences on PRs, it can be stated that increasing the number and diversity of data used in the classification performed on the entirety of PRs is necessary.

A review of the literature reveals that there are relatively few studies focusing on lesion classification on the entirety of PRs using the VGG16 model. In a study aimed at diagnosing jaw tumors, lesion classification was performed on a dataset consisting of 500 PRs, including 250 ameloblastomas and 250 odontogenic keratocysts, and the sensitivity and accuracy rates of the model in the study were reported as 81.8% and 83.1%, respectively [15]. In our study, which performed classification on the entirety of PRs and compared it with this literature, we observed that the diversity and localization of lesions, as well as the inconsistency in the number of data points based on lesion types, influenced our results.

In another classification study using the VGG16 model, a dataset consisting of PRs from 115 patients with nasopalatine canal cysts and 230 control group images without cysts was used, and the accuracy rate of the model in classification was reported as 88.4%. In the same study, the success rate of classification using the LeNet model was found to be 85.2% [27]. In our study, which classified radiopaque and radiolucent lesions on the entirety of PRs, the sensitivity and accuracy rates obtained using the VGG16 model were 98.8 and 68.4%, respectively. When classification was performed by cropping the lesion areas using the same model, these rates were found to be an average of 93 and 94%, respectively. In our study, which performed classification on the entirety of PRs and compared it with Ito et al. [27], we observed that the differences in the diversity and localization of lesions, as well as the imbalance in the number of data points according to lesion types, influenced our results.

In our study, the classification problem was approached with a variation by analyzing the PRs as a whole and by cropping the lesion-containing region from the same dataset. Additionally, these approaches were subjected to experiments under different scenarios as binary, three-class, and four-class classifications. During this process, CNN-based architectures, AlexNet, VGG16, and GoogleNet, were fine-tuned with our dataset. While the overall success in classification problems was achieved with the GoogleNet architecture, it was observed that the models produced more successful results when classifying cropped images compared to the entirety of PRs. In the literature, there is no study comparing these two different approaches together; however, based on the results of our study, we attribute the lower success rate of classification performed on the entirety of PRs to the size of the examined area, which is influenced by the complexity of surrounding anatomical structures.

In one study conducted on a dataset consisting of 412 PRs, only radiolucent lesions were detected with an accuracy rate of 75–77%. The lower success rate of the results obtained from this study compared to the detection of radiolucent lesions in the mandible was attributed to the low contrast between maxillary lesions and surrounding structures, as well as the superimposition of lesions by anatomical structures such as the maxillary sinus, nasal cavity, hard palate, inferior turbinate, and others, which makes them more challenging to interpret [16]. Additionally, a study reported that in some images, the extension of the maxillary sinus to the alveolar crest level could lead AI to mistakenly identify it as a lesion [28]. In line with the results of these studies, we observed in our study that superpositions caused by anatomical structures in PRs can affect the success of lesion detection. In the upper jaw, the low density exhibited by the maxillary sinus with septa formations, the nasal fossa, and airway arches that are particularly challenging for clinicians to distinguish has made the detection of radiolucent lesions more difficult, while the high density exhibited by the hard palate line, the hyoid bone superimposed on the mandible, vertebrae, and ghost images has complicated the detection of radiopaque lesions. This also affects the success of detecting not only radiolucent lesions but also radiopaque lesions in PRs. Therefore, in our study, without distinguishing between the maxilla and mandible, an average success rate of approximately 80% was achieved for detecting radiolucent lesions and approximately 70% for detecting radiopaque lesions, resulting in an overall average success rate of approximately 75%.

Yesiltepe et al. [29] in a study conducted using the GoogleNet architecture, aimed to detect lesions on 493 PRs with idiopathic osteosclerosis using the GoogleNet-Inceptionv2 model, achieving sensitivity, precision, and F1 score values of 0.88, 0.83, and 0.86, respectively. Radiopaque lesions other than idiopathic osteosclerosis were excluded from the study. In our study, however, all radiopaque lesions observed in the maxillofacial region were included in the classification, object detection, and segmentation problems, and similarly, the GoogleNet architecture was utilized for classification. It was determined that the proportional performance of the GoogleNet model for the classification of radiopaque–radiolucent lesions on the entirety of PRs showed sensitivity of 82.0%, precision of 70.5%, and an F1 score of 75.8%. In our study, in which we obtained results comparable to Yesiltepe et al. [29], we believe that the success is attributable to the performance of the model, despite the fact that the lesions comprising the dataset we used are not histopathologically uniform or single-density lesions, and the number of data points is not balanced according to the types of lesions.

In a study by Lee et al. [30], they utilized both PR and CBCT images as a dataset and evaluated the diagnosis and detection of odontogenic keratocysts, dentigerous cysts, and periapical cysts using a pre-trained deep CNN architecture derived from the GoogleNet-Inceptionv3 model. As a result of their research, the accuracy of the model was found to be 91.4% on CBCT images, while it was 84.6% on PRs. They attributed this high success rate on CBCT to the nature of the CBCT method, which allows for sectional examination in different planes without superposition.

Abdolali et al. [31] developed a method based on asymmetry analysis in the segmentation of lesions using a data set consisting of CBCT images of 97 patients with cystic lesions. As a result of their studies, they reported that due to factors such as the varying positions and sizes of cysts and their presentation with densities similar to surrounding tissues, traditional segmentation algorithms could produce a large number of false-positive pixels and exhibit poor segmentation performance. In our study, not only cystic images but also the segmentation of numerous lesions with varying densities were performed on PRs. Despite the large number of pixels in the images, which complicated the proper design of neural network models, a success rate of 72.1% was achieved with a limited dataset.

The three-dimensional volumetric calculation of lesions in the maxillofacial region offers many advantages during the evaluation process of lesions [32]. Accurate segmentation allows for the precise estimation of the localization and size of lesions [33]. In our study, which investigated with AI the localization of regions containing radiopaque and radiolucent lesions on PRs using bounding boxes and, more specifically, identifying the corresponding pixels in these areas, the YOLOv8 architecture capable of real-time detection was utilized. Ultimately, we achieved a success rate of 71.5% in lesion detection by drawing bounding boxes around objects and 72.1% in segmentation corresponding to pixel-level classification. Based on these results, we believe that segmentation processes performed with AI methods can facilitate volumetric detection of the lesioned area before surgical procedures and provide valuable insights for future studies in this field.

While radicular cysts and dentigerous cysts are among the most commonly observed lesions in the maxillofacial region, the occurrence of other pathological formations is less frequent, and this uneven distribution poses a challenge to the success of data sets obtained for studies [34]. Similarly, in our study, the distribution of radiopaque and radiolucent lesions in PRs obtained from archival materials was not equal. Lymph node calcifications and sialoliths, which produce radiopaque images and are rarely observed lesions, were also included in the study. These types of lesions are few in number and exhibit various localization patterns. Therefore, based on the results of our study, it can be stated that AI models achieved higher success in classifying, detecting, and segmenting radiolucent lesions compared to radiopaque lesions.

There are studies in the literature demonstrating that the high accuracy performance of AI algorithms in diagnosis and detection is equivalent to that of expert physicians [15, 35, 36]. However, these studies generally focus on the detection of specific types of lesions that clinicians do not find challenging to distinguish or address other issues unrelated to lesions, such as tooth numbering. In our study, however, we conducted research on a wide range of lesions that clinicians might find difficult to diagnose and that are not located in specific or standard positions. Additionally, the inclusion of both radiopaque and radiolucent lesions in the maxillofacial region highlights the comprehensive aspect of the study. By increasing the amount of data, we aim to develop more generalizable AI models. Thus, we believe that the automatic detection and classification of lesions and their characteristics in the maxillofacial region could become feasible in clinical practice.

Despite the promising results obtained in this study, several limitations inherent to the AI models and the study design must be acknowledged. First, the dataset, although larger than many previous studies, remains limited in terms of lesion diversity and distribution, particularly for rare radiopaque lesions such as sialoliths or idiopathic osteosclerosis. This imbalance may have introduced bias in model performance, favoring the detection and classification of more prevalent lesion types. Second, the inherent limitations of PRs, such as anatomical superimposition, geometric distortion, and lower resolution compared to advanced imaging modalities, pose significant challenges for AI models. These factors can lead to decreased performance, especially in detecting small or poorly defined lesions, or those located in anatomically complex regions such as the maxillary sinus or anterior nasal spine. Furthermore, the model-specific constraints were observed. While GoogleNet achieved the highest overall classification performance, its complex architecture may lead to increased computational demands and potential overfitting, particularly in smaller or less diverse datasets. VGG16, although demonstrating strong sensitivity for certain lesion types, showed lower accuracy in multi-class classification tasks, likely due to its limited capacity for hierarchical feature extraction. YOLOv8, despite its efficiency in real-time object detection, exhibited challenges in accurately detecting small or low-contrast lesions, suggesting limitations in its anchor box design and feature pyramid representation.

The experimental results and Grad-CAM analysis of all data showed that radiopaque and radiolucent lesions were not confused with each other. We can attribute the model’s erroneous results to the insufficient number of data to recognize the problem. Especially considering the wide variation in shape, pattern and location variation within the class, this study is considered to have promising initial performance. Moreover, we are hopeful that by increasing the number of data for both classes, we will be able to train models suitable for practical application in clinics.

Another critical limitation lies in the generalizability of the models. All experiments were conducted on a single-institution dataset acquired using a specific panoramic radiograph system, which may limit external applicability. The lack of external validation on datasets from different populations or imaging devices constrains the models’ generalizability across diverse clinical settings. Finally, while AI models demonstrate strong potential in automating lesion detection and classification, their integration into routine dental workflows requires further investigation, including prospective clinical validation, seamless software integration, and consideration of medico-legal responsibilities. These limitations underscore the need for continued research with larger, more diverse datasets, multi-institutional collaborations, and the development of explainable AI frameworks that can provide interpretable outputs to support clinical decision-making.

In conclusion, in his study, the presence of lesions in the maxillofacial region on PRs and their differentiation based on densities of image-producing lesions were evaluated using CNN-based DL algorithms AlexNet, VGG16, and GoogleNet, aiming to determine these models’ classification success. Additionally, the performance of the CNN-based YOLOv8 model in the detection and segmentation of radiopaque and radiolucent lesions was investigated. According to the study results, the ability of AI algorithms to identify lesions on PRs and classify them based on their densities is promising. This study aims to integrate AI models into hospital systems, enabling the effective application of state-of-the-art DL approaches in the clinical field and assisting dentists in lesion diagnosis.

The originality of this work stems from the problem itself and the comprehensive analysis applied to it, rather than from architectural innovation. A substantial effort was dedicated to the collection, curation, and meticulous labeling of the dataset, which required considerable domain expertise and time. Given the resource-intensive nature of this process, we made a deliberate decision to focus on fine-tuning well-established, high-performing architectures rather than developing new ones, to ensure that our results would be robust, reproducible, and directly comparable to the state-of-the-art.

## Data Availability

The data that support the findings of this study are not openly available due to reasons of sensitivity and are available from the corresponding author upon reasonable request.
